# Prize Dissertation on the Effects of Ardent Spirits

**Published:** 1828-10

**Authors:** 


18. *Prize Dissertation on the effects of Ardent Spirits.* The Massachusetts' Medical Society have offered a premium of FIFTY DOLLARS for the best Dissertation on the means of "*preventing and curing the habit of Intemperance.*" It is expected that those who become candidates will "*pcint out the circumstances in which the abandonment of the habitual use of stimulating drinks is dangerous; and, also investigate the effect of the use of wine and ardent spirits on the different organs and textures of the human body.*" The Dissertation must be sent in by the 15th of April 1829. The committee appointed to examine them, and award the premium, are Drs. John C. Warren, Zabdiel B. Adams, and John Ware.

The object of the Massachusetts' Society is of great interest, and we sincerely hope, that the effort may effectuate something useful to the profession and society at large. But where is the man who is qualified to extend our knowledge of the subject proposed? We do not doubt that candidates enough will press forward; but we hope the society will not content itself with sending forth a digest of common places.

